# Pilot Study to Reduce Added Salt on a University Canteen through the Use of an Innovative Dosage Equipment

**DOI:** 10.3390/foods11020149

**Published:** 2022-01-06

**Authors:** Ana Patrícia Faria, Patrícia Padrão, Olívia Pinho, Tânia Silva-Santos, Luís Oliveira, Sílvia Esteves, João Paulo Pereira, Pedro Graça, Pedro Moreira, Carla Gonçalves

**Affiliations:** 1 Faculty of Nutrition and Food Sciences, University of Porto, 4150-180 Porto, Portugal; up200806958@edu.fcna.up.pt (A.P.F.); patriciapadrao@fcna.up.pt (P.P.); oliviapinho@fcna.up.pt (O.P.); taniiasilvasantos@gmail.com (T.S.-S.); pedrograca@fcna.up.pt (P.G.); pedromoreira@fcna.up.pt (P.M.); 2 EPI Unit—Institute of Public Health, University of Porto, 4200-450 Porto, Portugal; 3 Laboratório Para a Investigação Integrativa e Translacional em Saúde Populacional (ITR), 4200-450 Porto, Portugal; 4 LAQV-REQUIMTE—Laboratory of Bromatology and Hydrology, Faculty of Pharmacy, University of Porto, 5000-801 Porto, Portugal; 5 INEGI—Instituto de Ciência e Inovação em Engenharia Mecânica e Engenharia Industrial, 4200-465 Porto, Portugal; loliveira@inegi.up.pt (L.O.); sesteves@inegi.up.pt (S.E.); jptp@inegi.up.pt (J.P.P.); 6 CIAFEL—Research Centre in Physical Activity, Health and Leisure, Faculty of Sport, University of Porto, 4200-450 Porto, Portugal; 7 CITAB—Centre for the Research and Technology of Agro-Environmental and Biological Sciences, University of Trás-os-Montes and Alto Douro, 5000-801 Vila Real, Portugal

**Keywords:** sodium, added salt, salt-reduction, consumer acceptance, food waste, canteens

## Abstract

Background: This study aims to demonstrate the practical application of an innovative easy-to-use equipment to dosage cooking salt, and evaluate the effectiveness in reducing 30% of the added salt in meals and the impact on consumer’s satisfaction and food waste. Methods: Two canteens from one public university where randomized in one control arm and one intervention arm. The first step was to evaluate the salt added to food through atomic emission spectrophotometry in both canteens, and the second step was to perform gradual reductions of up to 30% of cooking salt in the intervention canteen using the Salt Control-C (SC-C) equipment. Consumer acceptability was assessed through satisfaction questionnaires and food waste was evaluated by weighing. Results: The intervention canteen achieved to a reduction of more than 30% of added salt in soup (−34.3% per 100 g), fish dish (−41.1% per 100 g) and meat dish (−48.0% per 100 g), except for the vegetarian dish (6.1% per 100 g). There was no decrease in consumer satisfaction, with a significant satisfaction increase of 15.7% (*p* = 0.044) regarding the flavor of the main dish. Also, no significant differences were found in food waste. Conclusions: SC-C seems to be effective in reducing 30% of added salt levels in canteen meals, and may be a good strategy to control and reach adequate levels of added salt in meals served outside-the-home, promoting benefits to the individual’s health.

## 1. Introduction

Excessive salt consumption is a public health problem recognized worldwide as a risk factor for several health problems, such as hypertension-related cardiovascular diseases (CVD) [1,2,3,4,5,6] and other health complications such as chronic kidney disease, obesity, osteoporosis and gastric cancer [7]. Based on modelling data, 11 million deaths globally are associated with poor diet, 3 million of which are attributable to high sodium intakes which is also related to the loss of 70 million disability-adjusted life-years every year [8]. In Portugal, as well as in Europe, CVD are the leading cause of mortality [9,10]. In 2017, the inadequate eating habits of the Portuguese population were the fifth risk factor that most contributed to the loss of disability-adjusted life-years and mortality due to CVD, with high intake of sodium-rich foods among the top five dietary risk factors [11].

The World Health Organization (WHO) and policy governments around the world recognized and adopted reducing salt consumption by populations as a priority to reduce the prevalence of non-communicable diseases (NCD) [10,12,13,14,15,16,17,18]. In 2013, all WHO member states adhered to the goal of reducing 30% population salt intake until 2025 [19], with an ultimate goal of reaching the recommended daily maximum salt intake of 5 g/day (2 g/day of sodium) [7]. In the national strategy for reducing the salt consumption in portuguese population [18] the reduction of availability of foods with a high salt content and the monitoring of the supply (food and meals for sale) were considered imperative measures for public health.

In European countries, approximately 75–90% of salt intake is provided by the added salt in industrially processed foods and in cooked food, both prepared by restaurants/food concessionaires and at home, and only 10–25% occurs naturally in foods [20,21,22,23]. Indeed, the salt value in foods’ composition can vary considerably [20]. Gonçalves et al. analyzed the salt content in soups served in several public canteens in Portugal and, in addition to verifying a high average salt level (0.7 g/100 g of soup, i.e., 2.1 g of salt in a 300 g portion), they also found a high standard deviation, which demonstrates the existence of great variability in the levels of added salt [24]. In fact, most food handlers recognize that they use a random amount of salt based on personal flavor [25]. It makes it difficult for consumers to comply with dietary recommendations as the salt content of foods available in the market and restaurants is high and varies greatly.

Salt is a natural flavor enhancer in foods, but high levels of salt are naturally unpleasant and repulsive. However, frequent exposure to high levels may become pleasant, and people may develop a taste preference for foods with higher salt content [26,27,28,29,30]. Evidence suggests that the prefered level of salt in foods could be reduced over time, resulting in increased perceived salt intensity and decreased preference for salty foods [31,32,33,34]. One of the most important concerns of companies is the potential negative impact of these changes on consumers satisfaction and, consequently, impacting sales [25,31]. In that order, salt reduction interventions need to be gradually performed to be done without affecting consumer acceptability [35,36].

Since young people consume between a third and a half of meals at school or university canteens, this may be a key place for interventions that change the food environment, namely salt offer [37]. In this sense, the development of a quick and easy-to-use instrument to monitor and help control the salt added to food during the preparation of food in canteens could be part of the solution to reduce the population’s salt consumption.

In a systematic review, Mota et al. [38] concluded that intervention studies to reduce the salt content in meals provided by canteens are still insufficient and when reductions are gradual, there seems to be no negative impact on the acceptability of foods by the consumer. In this systematic review no intervention has used equipment to assist in salt dosing, so the present study is original and can bring new practical evidence in this field.

The aim of this study was to demonstrate the practical application of an innovative easy-to-use equipment of salt dosage, Salt Control-C (SC-C), and evaluate the effectiveness in reducing the added salt content of meals and the impact on consumer acceptability through an intervention in public university canteens.

## 2. Materials and Methods

### 2.1. Study Design

This study was conducted in two canteens (control canteen and intervention canteen) of a public university in northen Portugal, which serve exactly the same menus for lunch and dinner to students and workers from the university. The complete meal offered consists of vegetable soup, main dish (meat, fish or vegetarian), dessert and bread. The main dish includes the garnish (group of cereals, tubers, and/or pulses), the conduit (group of meat, fish and eggs, and/or pulses group or vegetable equivalent, as tofu or seitan), and the vegetables. 

**Figure 1:**
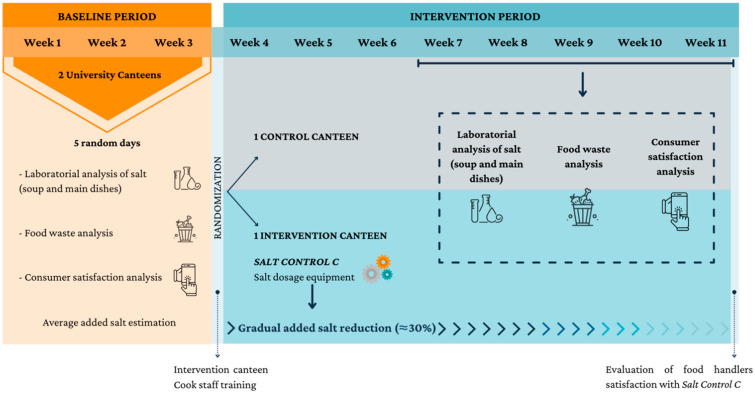
Flowchart with study design.

It is characterized as an experimental study divided into two consecutive steps: Step 1—baseline evaluation, and Step 2—intervention; performed between March and June of 2021. In the baseline step the salt added to food (soup and main dishes) was estimated in each canteen. During the intervention step, a gradual reduction of up to 30% of salt added to prepare soup and main dishes was carried out in the intervention canteen using the SC-C equipment, in relation to the average level of added salt obtained in baseline analysis. During the two steps of the study, consumer acceptability was assessed through satisfaction surveys and evaluation of plate food waste after mealtime (Figure 1).

#### 2.1.1. Step 1—Baseline Assessment

This occurred during the first three weeks, where the assessments were made in five random days on both canteens. The assessments consisted of the analysis of added salt in food (soup and main dishes), evaluation of consumer food waste and consumer satisfaction with meals. The menus information and the technical data of the corresponding recipes were also collected. 

The laboratorial salt analysis was performed to estimate the average usual level of salt added to prepare the meals in each canteen. This level of salt was used to program the SC-C equipment with the starting point to perform the gradual dosage reduction of salt dispensed to cook in the intervention canteen.

After the baseline period, the 2 canteens were randomly assigned, 1 was allocated to the intervention group and the other allocated to the control group.

#### 2.1.2. Step 2—Intervention

This step took place during eight consecutive weeks, where the SC-C was used in the intervention canteen to perform the salt dosage. The equipment was pre-programmed to implement a daily salt reduction until 30% less at the end of the intervention than the average of added salt content assessed in the baseline period. In the canteen allocated to the control group, the SC-C equipment was not implemented, and no training was given to the food handlers.

From week 7 until week 11, in both canteens, sodium levels were analyzed in five days in which the menus (soup and main dishes) coincided with the baseline period assessments, along with the measurement of food waste and consumer satisfaction questionnaires in the same days. Also during these weeks, extra meals were collected on another four random days (with the respective recipes technical data) to obtain a larger sampling of meals in order to assess the impact of the equipment on the added salt content of meals with more accuracy. 

The distribution of assessments over the last five weeks of intervention was as follows: week 7–8: three laboratorial salt analyses, two consumer satisfaction analyses and two food waste analyses; week 9–10: three laboratorial salt analyses, two consumer satisfaction analyses and two food waste analyses; week 11: three laboratorial salt analyses, one consumer satisfaction analysis and one food waste analysis.

### 2.2. Salt Content of Meals

Food samples from the soup and the three complete main dishes (vegetarian, meat and fish) were weighed and collected at each visit at the canteens. Only the edible parts were considered after deboning fish and meat. The samples were weighed in the kitchen and placed in properly coded plastic bags to be directly transported to the laboratory. They were mantained at refrigeration temperature (4 °C), for a maximum period of 24 h, until sample homogenization processment. The homogenization of the soup samples was made with a hand blender (Electric Co 450 W^®^), and the main dishes were mixed up with an electric food chopper (Moulinex 700W^®^) into a homogenous mass. The homogenized mass obtained was distributed on PTEE 60 mL containers and stored in a freezer (−18 °C) until it was used.

#### 2.2.1. Sodium Analysis

The evaluation of the sodium content of the collected meals was carried out by atomic emission spectrophotometry (AES) (flame photometry), the intern reference method to analyze sodium in food matrices [39], in the laboratory of the Faculty of Nutrition and Food Sciences of the University of Porto (FCNAUP).

After each food sample homogenization, 2 g was sampled and 2 mL of nitric acid was added. The mixture was shaken during 90 min to allow the food matrix to complete hydrolysis. Then, 20 mL of water was added, and the mixture was again homogenized using an electric homogenizer (Ultra Turrax blender T25, Sotel, Staufen, Germany). Volume was completed up to 40 mL and shaken for 30 min, followed by centrifugation (4000 rpm, 15 min; Labofuge 6000† Haerus model, Burladingen, Germany). Finally, 1 mL of aqueous supernatant was diluted up to 40 mL of deionized water before reading in the flame photometer (Model PFP7, JenWay, Staffordshire, UK). 

#### 2.2.2. Added Salt Levels

The total sodium values obtained in the analyses were converted into total salt (1 g of sodium corresponds to 2.5 g of cooking salt), and to obtain the values specifically of added salt in soup and main dishes the technical data of the recipes were consulted in order to calculate the value of the intrinsic salt of each ingredient according to data of the portuguese Food Composition Table or the food labels [40]. From the total salt value obtained by the analyses, the intrinsic salt value calculated was subtracted, obtaining the values of added salt for each soup and main dish. The added salt content was analyzed per 100 g of food and per portion.

### 2.3. Equipment to Dosage Cooking Salt: SC-C

The SC-C equipment (provisional patent INPI, No. 20211000015906) consists of a dosing device that provides doses of salt according to the age (children or adult) and the number of consumers. The prototype used in this study was made with a material compatible with food, and needs to be connected to the electrical current to operate.

**Figure 2:**
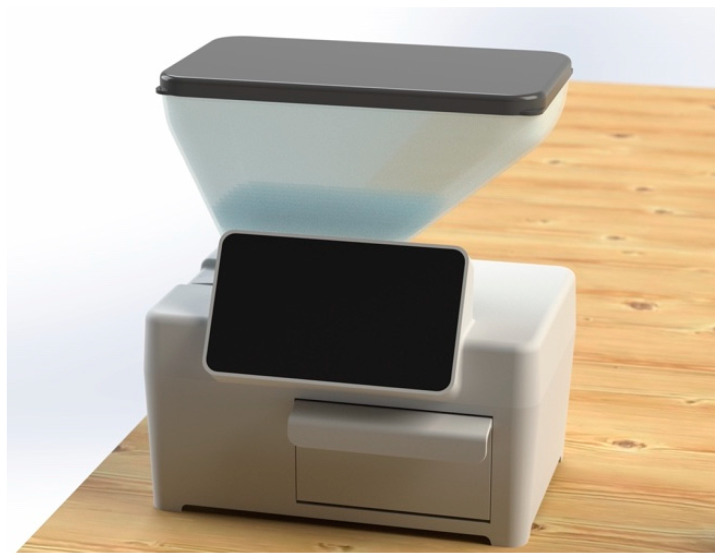
Salt Control-C equipment.

For soup, it dispenses salt according to the liters to be produced, and for main dishes it dispenses salt according to the number of servings to be cooked (Figure 2).

The equipment is available only for testing by researchers in controlled dietary studies, and not for commercial distribution. This prototype system was programmed to dispense a daily level of salt during the eight-week intervention period, with a 0.5% daily reduction in relation to the average baseline quantity of added salt in soup and main dishes obtained in the Step 1. 

Before starting the intervention step, food handlers in the intervention canteen received training about the use of the equipment. They were instructed to dosage the salt according to the liters of soup and the total number of main dishes to be cooked, and were responsible for personally managing the distribution of the dispensed salt to prepare the different recipes for the three main dishes (vegetarian, meat and fish). Also, they were informed that did not need to use the entire amount of salt dispensed when they considered it not necessary. During all the intervention period the daily maximum salt used for seasoning meals in the intervention canteen was exclusivelly the dispensed amount by the equipment. 

### 2.4. Satisfaction

#### 2.4.1. Consumers Satisfaction Questionnaires

In each evaluation day (5 days in the baseline period and 5 days in the intervention period), consumers were invited to answer a satisfaction questionnaire provided online in both canteens, by scanning QR Codes available on posters strategically placed after returning the food tray before the canteen exit.

In the questionnaire, the consent request for consumer participation was presented initially. It requested the sociodemographic information (sex and age) and the identification of the type of main dish consumed (meat, fish or vegetarian). Regarding meal satisfaction evaluation, seven questions about the degree of satisfaction were presented in a 1 to 5 score scale (from “totally dissatisfied” (1) to “totally satisfied” (5)). The first question was about the global satisfaction with meal, and the other six questions were specifically about the appreciation with the soup and with the main dish regarding global satisfaction, flavor and salt level satisfaction. 

#### 2.4.2. Consumers Food Waste

Food waste monitoring on consumers’ plates was also evaluated as a parameter of meal acceptability [41]. The measurement of food waste was carried out in each canteen kitchen pantry in the evaluation days (5 days in the baseline period and 5 days in the intervention period), through selective aggregate weighing [42]. This method allowed us to obtain an average waste value after removing all non-edible parts (such as bones, skin and fishbones). 

In each evaluation day, the weight of a soup model plate and the mean weight of the three main dish model plates were assessed (rejecting the weight of the non-edible parts of the food). Edible food waste in the consumers plates were separated and weighed by soup or main dish (three main dishes aggregated), in order to calculate the percentage of food waste for soup and main dish.

#### 2.4.3. Food Handlers Satisfaction Questionnaires

At the end of the intervention period, the cooks responsible for handling the SC-C answered a 5-level score scale satisfaction questionnaire (from “totally dissatisfied” (1) to “totally satisfied” (5)) regarding the experience of producing meals with the equipment for dosing the addition salt. The questionnaire presented eight satisfaction questions, particularly related to the control of the salt addition, the flavor of meals produced, the state of the equipment conservation, ease of use, promotion of eating habits, safety and hygiene conditions, and global satisfaction.

### 2.5. Data Analysis

Statistical analysis was performed using the statistical software IBM SPSS STATISTICS, version 26 (SPSS Inc., IBM, Chicago, IL, USA). Mean and standard deviations (SD) were used to characterize the study variables, namely food salt levels, food waste and consumer satisfaction. 

The mean or median from the five assessments (added salt and consumers satisfaction) carried out in the baseline period was computed and the same procedure was followed in the three assessments carried out in the week 11. The last week was chosen in order to compare the most extreme values of added salt reduction and correspondent consumer satisfaction. For the food waste, the comparison was performed with the median values of intervention week 10 and 11 assessments (*n* = 2), given that only one assessment was performed in the last week. The *t*-test for independent samples and the Mann–Whitney test were used to compare the values obtained between the assessments of the baseline period and the last week of intervention for each variable, as appropriate. 

Spearman and Pearson correlation coefficient was used to measure the degree of association between added salt levels in food with the food waste and the satisfaction levels of consumers. The null hypotesis was rejected when the critical significance level was less than 0.05.

### 2.6. Ethics Committee

The study was approved by the Ethics Committee of the Faculty of Nutrition and Food Sciences of the University of Porto.

## 3. Results

Table 1 shows the analysis for the added salt, food waste and consumer satisfaction in each canteen during the baseline and intervention steps of the study. In the control canteen it was found an increase in the avegare levels of added salt per portion of soup and the three main dishes, with a 135% significant increase in the soup portion at the end of the intervention (*p* = 0.025). In the intervention canteen, although the differences obtained were not statistically significant, it was found that there was a reduction in the levels of added salt in the soup (−34.4% per 100 g, and −48.6% per serving), in the meat dish (−48.0% per 100 g, and −51.8% per serving), and in the fish dish (−41.1% per 100 g, and −60.9% per serving). Regarding the vegetarian dish, there was a slight increase in added salt (6.1% per 100 g, and 1.2% per serving).

In terms of food waste, although there was an increase in the waste of soup and main dishes in both canteens (49.0% of total food in the control canteen, and 62.4% of total food in the intervention canteen), the differences found between the baseline period and the last two intervention weeks were not significant.

With regard to consumer satisfaction, in the control canteen there was a decrease in the percentage of satisfaction of all parameters evaluated, but the differences between the baseline period and the last week of intervention were not significant. In the intervention canteen there was an increase in the percentage of satisfaction of almost all the parameters evaluated, and for the satisfaction with the flavor of the main dish there was a significant increase of 15.7% (*p* = 0.044).

It was found two significant associations, one between added salt levels on soup per portion and global satisfaction with the meal (ρ = 0.133, *p* = 0.007), and other between total food waste and the added salt levels in soup per 100 g (ρ = −0.626, *p* = 0.003).

The two chefs responsible for handling the SC-C equipment rated between “satisfied” (3) and “totally satisfied” (5) all parameters evaluated in the satisfaction questionnaire, namelly, the questions about overall satisfaction and ease of using the device, one chef rated it as “totally satisfied” (5), and the other rated it as “very satisfied” (4), and regarding the flavor of the meals, both chefs rated it as “very satisfied” (4).

**Figure 3:**
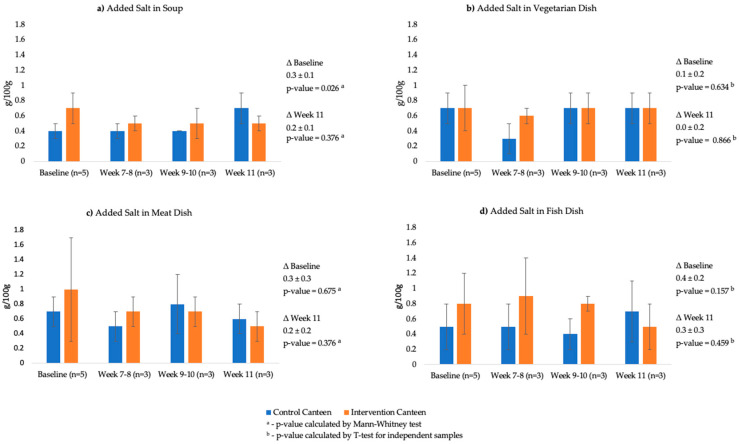
Values of added salt (g/100 g) over the course of the study in the control canteen and intervention canteen. Comparissons between canteens in the baseline period and in week 11. ^a^—*p*-value calculated by Mann–Whitney test; ^b^—*p*-value calculated by *t*-test for independent samples.

The Figure 3 presents the average values of added salt per 100 g in soup and main dishes in both canteens over the course of the study, and the differences between control and intervention canteen in the baseline period and in the last week of intervention. In the baseline period the intervention canteen showed higher average values of added salt per 100 g compared to the control canteen, but only with a significant difference in the soups (0.3 ± 0.1 g/100 g, *p* = 0.026).

The opposite was found in the last week (week 11) of the intervention period. The levels of added salt in the intervention canteen were lower than the control canteen, with the exception of the vegetarian dish where the levels of added salt remained very close in the two canteens, however without significant differences.

It was also observed that the amount of salt added to the dishes, compared to the total salt values obtained in the laboratorial analyses, corresponded to an average of 94% of the salt in the soup as well as in the vegetarian dish, 88% in the fish dish and 84% in the meat dish. The results of the total salt values per dish obtained through the chemical analysis are available in Appendix A.

## 4. Discussion

This pilot study shows that this innovative equipment that dose the amount of salt available to prepare and cook meals in canteens is effective in the reduction of around 30% of added salt. Although no statistically significant differences were obtained, that may be due to the small sample size, and we verified an added salt reduction superior to 30% in the last intervention week for soup, fish and meat dishes. However, the salt levels in the vegetarian dish remained close to the baseline mean. The cooks demonstrated a preference for managing the salt to make greater reductions in fish and meat dishes, keeping the salt levels in the vegetarian dish similar to baseline levels. It could be due to the fact that these dishes are constituted only by vegetable ingredients, which themselves contain lower levels of intrinsic salt, and tend to have the unpleasant natural bitterness that salt could mask and increase saltiness and sweetness flavors [43,44]. This event may also be caused by the cooks’ lack of practice and confidence in the preparation of vegetarian meals [45,46], but more studies are needed to explore this assumption regarding the possible difficulty of reducing salt in vegetarian dishes.

Regarding the evaluation of consumer acceptability throughout the intervention, there was no decrease in meal satisfaction. On the contrary, there was a significant increase of 15.7% in satisfaction level regarding the flavor of the main dish. Other studies conducted in Portugal and other countries, with groups of different ages, have reported reductions between 20% to 30% salt content in food without affecting consumer acceptability [36,47,48]. 

No significant differences were found in the food waste in consumers’ plates between baseline and the last two intervention evaluations. A value of 10% has been pointed in the literature as a benchmark for acceptable food waste levels, and the limit of 5% is being considered optimal service performance [49,50]. The control canteen showed mean percentages of total waste throughout the study ranging from 7.5% to 11.9%. Similar food waste percentages were obtained in a study carried out by Aires C. et al. in a Portuguese university canteen [51]. On the other hand, the results of the baseline evaluation in the intervention canteen showed much lower levels of food waste, matching the optimal levels (under 5%) throughout the complete study. Although no significant differences were found on consumers satisfaction and food waste, and the levels observed in the intervention canteen can be considered very satisfactory, soup waste increased on average 167.6% between the mean baseline evaluations and the last two intervention evaluations. Also, a significant negative moderate correlation was observed between the soup salt content and total food waste, suggesting that consumers may prefer soup with higher salt levels. Once again, we observed a possible tendency towards a preference for higher salt levels in dishes made up exclusively of vegetables as soup. A possible strategy to stimulate lower levels of salt in soup and other exclusive vegetable dishes may be the improvement of recipes and food education sessions for food handlers to sensitize and encourage the use of ingredients with a high impact on flavor such as fresh herbs, garlic, onion, and spices to help masking low salt levels, and offering beneficial properties for human health at the same time [44,52].

Another important result was the contribution of this equipment to the standardization of the added salt to meals. Indeed, high standard deviation (SD) values of added salt were observed, especially in the intervention canteen in the five baseline assessments. During the entire intervention period of salt dosage with SC-C, as expected, a smaller SD was observed in the salt added to the dishes (SD off added salt per portion: 0.4 g in soup; 0.7 g in vegetarian; 0.8 g in meat; 1.2 g in fish). Also, between the baseline period and the last intervention week in the control canteen, there was a significant difference, of more than twice, on the average levels of added salt in soup. The values were very heterogeneous, showing no pattern of added salt between different collection days and between canteens. This is probably because the salt added to food may vary according to the cooks’ intrapersonal and interpersonal taste and food preparation practices. Similar results of the high variability between meals salt content were observed by Barbosa et al. in a study conducted in seven Portuguese university canteens [53]. 

A practical standardization strategy of adding salt in food preparation, that respects the daily salt recommendations, is of great importance to stabilize and control the salt content throughout the days and across canteens. A research with Portuguese food handlers showed that many were aware of the maximum salt intake recommendations and the health problems associated with excessive salt consumption. They reported being willing to reduce the salt content of foods produced. However, the greatest difficulty in reducing salt pointed out was the consumers’ opinion and the knowledge of food handlers of how to proceed [25]. In this pilot study, particular attention was paid to food handlers, providing them training and initial mentoring in the use of the equipment. Although the cooks’ sample was very small, in the satisfaction questionnaire answered, they showed good satisfaction levels with SC-C equipment to carry out the dosage of cooking salt.

Since WHO recommends a maximum daily salt intake of less then 5 g [7], the salt levels provided by canteens can be considered excessive. The average total salt content per complete meal (soup and main dish) of both canteens, prepared without the intervention of the SC-C equipment, was 5.9 ± 1.9 g/portion, which corresponds to 118% of the maximum recommended daily salt intake. Even in the last intervention week, a complete meal offered in the intervention canteen provided, on average, a total salt value of 3.9 ± 1.1 g/portion, which corresponds to 77% of the recommended daily salt intake. In both canteens, the average salt levels in the soup and main dishes obtained in the set of days of the baseline period are similar, or even higher, than others reported in the literature [24,47,53,54]. In the study carried out in Portuguese university canteens, Gonçalves et al. observed that the average salt content of one meal (soup and main dish) reached about 53% of the recommended daily intake [53]. Another two research articles conducted in Portugal obtained range values of added salt per 100 g on soup in nursing homes, kindergartens and elementary schools similar to those we obtained [24,47]. 

Salt reductions must be performed without negatively impacting consumers’ salt and hedonic perceptions because, for individuals who are used to tasting high levels of salt, its sudden decrease may cause food rejection [31,32,44,55]. It is especially relevant for food companies as these types of interventions may impact sales. Thus, small gradual reductions of added salt might be a valuable strategy to reduce salt intake, and this study reinforces 30% as a feasible benchmark for reduction interventions. 

The dosage with SC-C equipment is a practical intervention comprising simple training to food handlers that may positively impact patterning and reductions in added salt levels. It can be easily implemented in different settings and similar institutions, and the results of this study may help support the reproduction of this type of intervention. Many workplaces, schools or social institutions provide daily meals and thus have the potential to provide options and induce healthy food consumption for their consumers [56]. Geaney et al. have shown that structured catering initiatives in the public sector can reduce dietary salt intakes [57]. Reynoso et al. performed an intervention study of a 20% added salt gradual reduction on a working food concessionaire in Peru, without affecting consumers’ food acceptability, and demonstrated a significant positive impact on reducing customers’ blood pressure. 

In terms of public health, it would be desirable that consumers exposed to meals outside the home could benefit from meals that provide adequate salt levels and make informed choices. Longer studies are needed to assess the long-term effects and evaluate the implementation of greater added salt reductions. 

Due to the pilot experimental design, this study has several limitations that include the possibility of making inaccurate predictions or assumptions on the basis of pilot data, as well as problems related to the small sample and small assessments that could be reflected in uncertainties in the results. In addition, the small number of consumers in canteens occured because many of the classes were taking place online due to the COVID-19 pandemic restrictions. In future larger studies, the number of assessments should be increased consistently throughout the study for all the outcomes.

The consumers were unaware of the salt reduction strategy, so we consider it could not bias results. The innovative proposal, the experimental nature with a control and intervention group selected randomly, and the reference laboratorial methodology used to accurately detect salt levels in foods represent the main strengths of this pilot study.

## 5. Conclusions

The use of the SC-C equipment to perform the dosage of cooking salt, programmed for a gradual reduction of 30% over the eight weeks of intervention, seems to be effective in reducing the levels of salt in the soup (−34.3% per 100 g), meat dish (−48.0% per 100 g) and fish dish (−41.1% per 100 g). However, in the vegetarian dish there was no decrease in the salt content. There was a significant increase of 17.5% in satisfaction level with the taste of main dish, and no significant impact in terms of food waste on consumers’ plates was observed.

This work presents data that show a wide variation in the values of added salt within and between canteens. It has also been found that consumers of university canteens can easily exceed the WHO maximum daily salt intake recommendations. 

This equipment is intended to help food handlers to control and approximate the added amount of cooking salt to values that respects the WHO recommendations through a practical procedure. Similar interventions should be replicated in similar and other contexts as they may positively impact the efforts to reach adequate levels of added salt in meals served outside the home and thus promote benefits to individuals’ health.

## Figures and Tables

**Table 1:** Added salt, food waste and consumer satisfaction evaluations per canteen over the course of the study.

			Baseline Week 1–3	Week 7–8	Week 9–10	Week 11	Δ% Baseline vs. Week 11	*p*-Value
Control Canteen								
		Samples (n)	5	3	3	3		
Added Salt Mean ± SD	Soup	g/100 g	0.4 ± 0.1	0.4 ± 0.1	0.4 ± 0.0	0.7 ± 0.2	66.8	0.050 ^a^
		g/portion	1.0 ± 0.4	1.0 ± 0.3	1.3 ± 0.2	2.3 ± 0.4	135.0	0.025 ^a^
	Vegetarian	g/100 g	0.7 ± 0.2	0.3 ± 0.2	0.7 ± 0.2	0.7 ± 0.2	−9.9	0.633 ^b^
		g/portion	2.4 ± 0.7	1.5 ± 0.9	3.0 ± 0.7	3.1 ± 1.0	32.0	0.683 ^b^
	Meat	g/100 g	0.7 ± 0.2	0.5 ± 0.2	0.8 ± 0.4	0.6 ± 0.2	−1.9	1.000 ^a^
		g/portion	2.3 ± 0.8	2.1 ± 1.0	3.0 ± 1.1	2.6 ± 1.3	12.7	0.880 ^a^
	Fish	g/100 g	0.5 ± 0.3	0.5 ± 0.3	0.4 ± 0.2	0.7 ± 0.4	59.4	0.315 ^b^
		g/portion	1.3 ± 0.7	1.8 ± 1.1	1.7 ± 0.6	2.9 ± 1.7	117.9	0.297 ^a^
Food waste Mean ± SD	Consumers (n)		71	84	66	28		
	Soup	%	7.3 ± 5.0	5.8 ± 8.2	11.7 ± 1.1	8.1	30.2 *	0.585 *^,a^
	Main dish		7.5 ± 5.9	10.5 ± 2.5	12.0 ± 1.1	12.5	69.1 *	0.245 *^,a^
	Total		7.5 ± 4.5	8.6 ± 4.8	11.9 ± 0.1	10.5	49.0 *	0.245 *^,a^
Consumers satisfaction (score 1–5) Mean ± SD	Global							
	answers (n)		36	32	40	7	−1.0	0.892 ^b^
	evaluation		4.5 ± 0.8	4.1 ± 0.8	4.2 ± 0.9	4.4 ± 0.5		
	Soup—global							
	answers (n)		31	30	39	7	−6.6	0.452 ^b^
	evaluation		4.1 ± 0.8	3.9 ± 0.9	3.7 ± 1.2	3.9 ± 1.1		
	Soup—flavor							
	answers (n)		31	28	37	7	−13.6	0.148 ^b^
	evaluation		4.1 ± 0.9	3.8 ± 1.1	3.6 ± 1.3	3.6 ± 1.0		
	Soup—salt							
	answers (n)		31	28	37	7	−7.5	0.394 ^b^
	evaluation		4.3 ± 0.9	3.6 ± 1.2	3.9 ± 1.3	4.0 ± 0.8		
	Main dish—global							
	answers (n)		31	31	38	7	−9.8	0.271 ^b^
	evaluation		4.3 ± 0.9	3.7 ± 1.0	4.0 ± 0.8	3.9 ± 1.1		
	Main dish—flavor							
	answers (n)		31	29	36	7	−10.4	0.230 ^b^
	evaluation		4.3 ± 0.9	3.7 ± 1.1	4.0 ± 0.8	3.9 ± 0.9		
	Main dish—salt							
	answers (n)		31	29	36	7	−13.0	0.336 ^b^
	evaluation		4.5 ± 0.8	3.6 ± 1.1	4.0 ± 1.0	3.9 ± 1.5		
Intervention Canteen								
		Samples (n)	5	3	3	3		
Added Salt Mean ± SD	Soup	g/100 g	0.7 ± 0.2	0.5 ± 0.1	0.5 ± 0.2	0.5 ± 0.1	−34.3	0.131 ^a^
		g/portion	2.4 ± 0.7	1.2 ± 0.4	1.3 ± 0.6	1.2 ± 0.3	−48.6	0.053 ^a^
	Vegetarian	g/100 g	0.7 ± 0.3	0.6 ± 0.1	0.7 ± 0.2	0.7 ± 0.2	6.1	0.855 ^b^
		g/portion	2.8 ± 1.4	2.3 ± 0.4	3.0 ± 1.0	2.9 ± 0.6	1.2	0.906 ^b^
	Meat	g/100 g	1.0 ± 0.7	0.7 ± 0.2	0.7 ± 0.2	0.5 ± 0.2	−48.0	0.230 ^a^
		g/portion	3.8 ± 2.4	2.8 ± 1.2	2.0 ± 0.5	1.8 ± 0.4	−51.8	0.230 ^a^
	Fish	g/100 g	0.8 ± 0.4	0.9 ± 0.5	0.8 ± 0.1	0.5 ± 0.3	−41.1	0.284 ^b^
		g/portion	3.4 ± 2.2	3.0 ± 1.6	2.5 ± 0.8	1.3 ± 0.8	−60.9	0.297 ^a^
Food waste Mean ± SD	Consumers (n)		36	438	312	123		
	Soup	%	1.4 ± 2.5	3.9 ± 2.2	0.4 ± 0.4	3.9	167.6 *	0.252 *^,a^
	Main dish		3.0 ± 2.1	2.1 ± 0.2	1.8 ± 1.7	3.9	17.5 *	0.699 *^,a^
	Total		2.2 ± 1.8	2.8 ± 0.8	2.7 ± 0.9	3.9	62.4 *	0.699 *^,a^
Consumers satisfaction (score 1–5) Mean ± SD	Global							
	answers (n)		28	96	141	46	2.9	0.607 ^b^
	evaluation		4.0 ± 0.9	3.7 ± 0.9	3.8 ± 0.9	4.2 ± 1.0		
	Soup—global							
	answers (n)		28	94	132	44	3.0	0.660 ^b^
	evaluation		3.9 ± 1.1	3.2 ± 1.1	3.4 ± 1.1	4.0 ± 1.1		
	Soup—flavor							
	answers (n)		27	88	123	44	6.8	0.354 ^b^
	evaluation		3.7 ± 1.2	3.0 ± 1.0	3.4 ± 1.0	4.0 ± 1.0		
	Soup—salt							
	answers (n)		27	88	124	44	12.2	0.098 ^b^
	evaluation		3.6 ± 1.1	3.0 ± 1.3	3.5 ± 1.1	4.1 ± 1.1		
	Main dish—global							
	answers (n)		27	95	140	46	−3.3	0.611 ^b^
	evaluation		3.8 ± 0.9	3.3 ± 0.9	3.5 ± 1.0	3.7 ± 1.1		
	Main dish—flavor							
	answers (n)		26	90	136	45	15.7	0.044 ^b^
	evaluation		3.4 ± 1.2	3.3 ± 1.0	3.5 ± 1.1	4.0 ± 1.0		
	Main dish—salt							
	answers (n)		26	90	136	45	7.4	0.299 ^b^
	evaluation		3.6 ± 1.1	3.4 ± 1.0	3.6 ± 1.1	3.9 ± 1.0		

^a^ *p*-value calculated by Mann–Whitney test between baseline period and intervention week 11 evaluations; ^b^ *p*-value calculated by *t*-test for independent samples, between baseline period and intervention week 11 evaluations; * Comparisson between the baseline period evaluations (*n* = 5) and week 10 and 11 evaluations (*n* = 2).

## Data Availability

The authors will make the data available by correspondent request.

## Appendix A

**Table A1:** Total salt content of the soup and main dishes per canteen over the course of the study (total sodium by chemical analisis).

		Mean ± SD	Baseline Week 1–3 (*n* = 5)	Week 7–8 (*n* = 3)	Week 9–10 (*n* = 3)	Week 11 (*n* = 3)
Control Canteen	Soup	g/100 g	0.4 ± 0.1	0.4 ± 0.1	0.5 ± 0.0	0.7 ± 0.2
		g/portion	1.1 ± 0.4	1.1 ± 0.3	1.4 ± 0.2	2.4 ± 0.5
	Vegetarian	g/100 g	0.8 ± 0.2	0.4 ± 0.2	0.7 ± 0.2	0.8 ± 0.2
		g/portion	2.5 ± 0.7	1.6 ± 0.9	3.1 ± 0.7	3.6 ± 0.7
	Meat	g/100 g	0.8 ± 0.3	0.6 ± 0.2	0.9 ± 0.4	0.9 ± 0.1
		g/portion	2.7 ± 1.0	2.6 ± 1.2	3.4 ± 1.2	3.5 ± 1.0
	Fish	g/100 g	0.5 ± 0.3	0.6 ± 0.3	0.5 ± 0.2	0.9 ± 0.3
		g/portion	1.5 ± 0.8	2.1 ± 1.2	1.9 ± 0.6	3.4 ± 1.1
Intervention Canteen	Soup	g/100 g	0.8 ± 0.2	0.5 ± 0.1	0.5 ± 0.2	0.5 ± 0.1
		g/portion	2.5 ± 0.7	1.3 ± 0.4	1.4 ± 0.6	1.3 ± 0.3
	Vegetarian	g/100 g	0.7 ± 0.3	0.6 ± 0.1	0.7 ± 0.2	0.8 ± 0.3
		g/portion	2.9 ± 1.4	2.4 ± 0.3	3.1 ± 1.1	3.2 ± 0.8
	Meat	g/100 g	1.1 ± 0.7	0.8 ± 0.3	0.8 ± 0.1	0.7 ± 0.2
		g/portion	4.3 ± 2.4	3.3 ± 1.6	2.3 ± 0.6	2.8 ± 1.1
	Fish	g/100 g	0.9 ± 0.4	1.0 ± 0.6	0.8 ± 0.1	0.6 ± 0.1
		g/portion	3.6 ± 2.1	3.3 ± 1.7	2.7 ± 0.8	1.6 ± 0.5
